# Clinical and pathological factors associated with recurrence after radical cystectomy for bladder cancer: A single-center retrospective study

**DOI:** 10.12669/pjms.42.4.14997

**Published:** 2026-04

**Authors:** Nadeem Bin Nusrat, Sarmad Imtiaz, Asadullah Aslam, Assad ur Rehman, Shujah Muhammad, Nauman Zafar, Abdullah Muhammad Ashfaq, Hassan Arshad

**Affiliations:** 1 Nadeem Bin Nusrat, FRCS Ed (intercollegiate specialty board of Urology), CCST, Department of Urology, Pakistan Kidney, Liver Institute and Research Center (PKLI & RC), Lahore, Pakistan; 2 Sarmad Imtiaz, MS Urology, Department of Urology, Pakistan Kidney, Liver Institute and Research Center (PKLI & RC), Lahore, Pakistan; 3 Asadullah Aslam, FCPS Urology, Department of Urology, Pakistan Kidney, Liver Institute and Research Center (PKLI & RC), Lahore, Pakistan; 4 Assad ur Rehman, FCPS Urology, Department of Urology, Pakistan Kidney, Liver Institute and Research Center (PKLI & RC), Lahore, Pakistan; 5 Shujah Muhammad, FCPS Urology, Department of Urology, Pakistan Kidney, Liver Institute and Research Center (PKLI & RC), Lahore, Pakistan; 6 Nauman Zafar, MRCS Urology, Department of Urology, Pakistan Kidney, Liver Institute and Research Center (PKLI & RC), Lahore, Pakistan; 7 Abdullah Muhammad Ashfaq, MBBS, FCPS Part I, Department of Urology, Pakistan Kidney, Liver Institute and Research Center (PKLI & RC), Lahore, Pakistan; 8 Hassan Arshad, MBBS, Department of Urology, Pakistan Kidney, Liver Institute and Research Center (PKLI & RC), Lahore, Pakistan

**Keywords:** Bladder cancer, Recurrence, Radical cystectomy

## Abstract

**Background and Objective:**

Radical cystectomy (RC) is standard treatment for muscle-invasive bladder cancer (MIBC); however, disease recurrence remains a major challenge. This study aimed to describe recurrence patterns and explore clinical and pathological factors associated with early recurrence post-RC.

**Methodology:**

This retrospective cohort study included adult patients with histologically confirmed bladder cancer who underwent RC at the Pakistan Kidney and Liver Institute & Research Center, Lahore, between February 2023 and August 2025. Demographic, pathological, treatment, and follow-up data were extracted. Recurrence status was available for 40 patients and was analyzed using descriptive statistics and univariate analyses.

**Results:**

Among 70 patients, follow-up sufficient to evaluate recurrence was available for 40 (57.1%). Recurrence occurred in nine patients (9/40, 22.5%) during the available follow-up period. Mean time to recurrence among affected patients was 5.0 ± 2.7 months. Histological subtype showed a possible signal with recurrence, with squamous cell carcinoma appearing more frequently among recurrence cases (p = 0.019); however, this observation was based on a very small number of patients and should be interpreted with caution. Other demographic, tumor-related, and treatment variables were not significantly associated with recurrence. Follow-up duration was varied and shorter among recurrence cases compared with non-recurrence cases, which may establish detection bias.

**Conclusion:**

In this single-center cohort, early recurrence after radical cystectomy was observed in a subset of patients; however, the small sample size, short follow-up duration, and substantial loss to follow-up limit reliable identification of predictors. Larger prospective studies are needed to characterize recurrence patterns post-RC.

## INTRODUCTION

Bladder cancer is one of the most common cancers of the urinary system, and its burden is enormous. Muscle-invasive bladder cancer (MIBC) makes up 25-30% of newly diagnosed bladder cancer cases, and its burden is significant in terms of morbidity and mortality.^1^,^2^ Radical cystectomy with pelvic lymph node dissection is the main treatment for MIBC and some high-risk non-muscle-invasive bladder cancer.^3^,^4^

The problem of cancer recurrence following radical cystectomy is still a great concern. Recurrence may occur locally within the pelvis or as distant metastatic disease, often within the first two years following surgery. Previous studies have identified several factors associated with recurrence, including pathological stage, lymph node involvement, tumor grade, histological variants, and response to neoadjuvant chemotherapy.^5^-^7^

However, most evidence originates from Western populations, 8 and data from South Asian centers remain limited. Differences in disease presentation, access to care, and follow-up patterns may influence oncologic outcomes in this region. Therefore, real-world institutional data may provide useful insights into recurrence patterns following radical cystectomy. 9,10

The present study aimed to describe recurrence patterns and explore clinical and pathological factors potentially associated with early recurrence following radical cystectomy at a tertiary care center in Pakistan.

## METHODOLOGY

This retrospective observational cohort study was conducted at the Department of Urology, Pakistan Kidney and Liver Institute & Research Center (PKLI & RC), Lahore, a tertiary care teaching hospital with advanced robotic and open surgical facilities.

### Ethical approval:

It was obtained from the Institutional Review Board of PKLI & RC (PKLI-IRB/AP/0069/2025; July 29, 2025). Written informed consent was waived due to the retrospective design and use of anonymized clinical data.

Adult patients (≥18 years) with histologically confirmed bladder cancer who underwent initial transurethral resection of bladder tumor (TURBT), with or without intravesical therapy, were included between February 1^st^ 2023 to August 28^th^ 2025. Patients with metastatic disease at the time of diagnosis or those who were mainly treated outside PKLI & RC were excluded. A non-probability purposive sampling technique was used, and all patients who met the criteria were included in the study without any calculation of sample size.

### Data collection:

Data collection was done retrospectively from electronic patient data, surgical notes, histopathology results, radiology results, and follow-up results. The data collected consisted of demographic information (age, gender, smoking status, and comorbidities), tumor-related information (size, number, and site of tumor, pathological grade and stage, and tumor margins), treatment-related information (extent of TURBT, perioperative complications, intravesical treatment modalities, and treatment responses), and surveillance results (information from cystoscopy and imaging modalities). The main outcome measure was tumor recurrence, which was histologically or radiologically proven recurrence during follow-up and was a binary variable (yes/no), along with time to recurrence. Patients were followed postoperatively according to institutional surveillance practices. Follow-up evaluations included routine clinical assessment, imaging studies, and imaging-based surveillance, with cystoscopic evaluation when clinically indicated. Follow-up information was obtained from outpatient clinic records and electronic medical records.

The duration of follow-up varied among patients because of differences in the timing of surgery and availability of follow-up data. The last patient included in the cohort underwent surgery on 28 August 2025, and follow-up data were available up to the time of data extraction. Recurrence status could be determined for 40 patients with available follow-up information, and these patients were included in the recurrence analysis. Patients without sufficient follow-up data were excluded from outcome analyses.

### Statistical Analysis:

Data extraction was performed by two trained postgraduate residents using a standardized abstraction form under supervision of the principal investigator. Data were entered into IBM SPSS Statistics (version 27) database. Associations between recurrence and potential predictors were explored using chi-square or Fisher’s exact test for categorical variables and independent-samples t-test or Mann–Whitney U test for continuous variables. Because of the small number of recurrence events (n = 9), multivariable regression modeling was not considered statistically reliable and was therefore not included in the final analysis. A p-value < 0.05 was considered statistically significant.

## RESULTS

A total of 70 patients with bladder cancer who underwent radical cystectomy at PKLI & RC were included in the baseline cohort. The majority were male (54/70, 77.1%). Previous bladder surgery was common (60/70, 85.7%), and most tumors were high grade (56/70, 80.0%) with muscle-invasive disease (T2–T4: 42/70, 60.0%). Radical cystectomy with ileal conduit was performed in all patients.

Follow-up information sufficient to assess recurrence was available for 40 patients, constituting the analytical cohort. Of these, nine patients experienced recurrence, while 31 did not. The remaining 30 patients had missing recurrence data or were lost to follow-up and were excluded from recurrence analyses. Baseline clinical, pathological, treatment, and follow-up characteristics of the full cohort are summarized in Table-I and II

**Table-I:** Baseline Demographic and Clinical Characteristics of Patients Undergoing Radical Cystectomy (N = 70).

Variable	n (%), * mean ± SD
Age (years), mean ± SD	57.1 ± 10.2
Male gender	54 (77.1)
Smoking history	36 (51.4)
Body mass index (kg/m²)	26.4 ± 4.3*
Duration of symptoms (months)	12.3 ± 18.6*
***ECOG performance status***	
ECOG 0	55 (78.6)
ECOG 1–2	15 (21.4)
Diabetes mellitus	8 (11.4)
Hypertension	18 (25.7)

Data are presented as n (%). For several variables, data were missing due to incomplete documentation or loss to follow-up; therefore, denominators may vary across variables and may not sum to the total cohort size (N = 70).

**Table-II:** Tumor and Pathological Characteristics of the Cohort (N = 70).

Variable	n (%)
***Presenting symptoms***	
Hematuria	68 (97.1)
Dysuria	36 (51.4)
Urinary frequency	18 (25.7)
Urgency	13 (18.6)
Pain	8 (11.4)
Lower abdominal mass	8 (11.4)
***Tumor location on TURBT***	
Multiple locations	23 (32.9)
Lateral wall	10 (14.3)
Anterior wall	1 (1.4)
***Number of tumors***	
Single	3 (4.3)
Multiple	26 (37.1)
***Histological type***	
Urothelial carcinoma	57 (81.4)
Squamous cell carcinoma	4 (5.7)
***Tumor grade***	
High grade	56 (80.0)
Low grade	7 (10.0)
***Pathological stage***	
T1	21 (30.0)
T2	35 (50.0)
T3	4 (5.7)
T4	3 (4.3)
***Imaging modality***	
CT scan	46 (65.7)
MRI	16 (22.9)
***Lymphadenopathy on imaging***	
Present	33 (47.1)
Absent	29 (41.4)
***Surrounding tissue invasion***	
Muscle	20 (28.6)
Perivesical tissue	16 (22.9)
Adjacent organs	18 (25.7)
None	8 (11.4)
***Ureteral invasion***	
Present	34 (48.6)
Absent	29 (41.4)

Data are presented as n (%). For several variables, data were missing due to incomplete documentation or loss to follow-up; therefore, denominators may vary across variables and may not sum to the total cohort size (N = 70).

Among patients with available follow-up (n = 40), recurrence was observed in nine patients (22.5%). Recurrence sites were documented and identified as occurring in the ureter, lymph nodes, and distant sites. The length of follow-up within the analytical cohort was brief and varied, with a median length of only three months. The patients who had recurrence had a mean length of time until recurrence of 5.0 ± 2.7 months, as identified in nine patients. The patients without recurrence had a significantly longer mean length of follow-up at 17.8 ± 14.7 months. Table-III

**Table-III:** Treatment Characteristics and Follow-Up Outcomes (N = 70).

Variable	n (%)
Initial TURBT performed	62 (88.6)
***TURBT complexity***	
Routine TURBT	31 (44.3)
Complex TURBT	13 (18.6)
***Completeness of TURBT***	
Complete	50 (71.4)
Incomplete	20 (28.6)
***Intraoperative complications***	
Bleeding	23 (32.9)
Bladder perforation	15 (21.4)
None	32 (45.7)
***Postoperative complications***	
Present	16 (22.9)
Absent	54 (77.1)
***Intravesical therapy***	
BCG therapy	5 (7.1)
Intravesical chemotherapy	1 (1.4)
***Neoadjuvant chemotherapy***	
Received	26 (37.1)
Gemcitabine + carboplatin	9 (12.9)
***Radiotherapy***	
Received	12 (17.1)
***Definitive surgical treatment***	
Radical cystectomy + ileal conduit	70 (100.0)
***Treatment-related complications***	
Any complication	39 (55.7)
Infection	15 (21.4)
Stomal issues	6 (8.6)
Follow-up available	40 (57.1)
***Recurrence (patients with available follow-up, n = 40)***	
Yes	9 (12.9)
No	31 (44.3)
***Documented recurrence sites***	
Ureter	1 (1.4)
Pelvic lymph nodes	2 (2.9)
Distant metastasis	1 (1.4)

Data are presented as n (%). For several variables, data were missing due to incomplete documentation or loss to follow-up; therefore, denominators may vary across variables and may not sum to the total cohort size (N = 70).

Univariate analyses were restricted to the analytical cohort (n = 40) and are presented in Table-IV. Most demographic, clinical, surgical, pathological, and treatment-related variables showed no statistically significant association with recurrence.

**Table-IV:** Univariate Analysis of Factors Associated with Recurrence (Analytical Cohort n = 40)

Variable	Recurrence (Yes) n = 9	Recurrence (No) n = 31	p-value
Age (years), mean ± SD	56.0 ± 11.5	57.5 ± 9.8	0.704
Male gender	9 (100)	25 (80.6)	0.152†
Smoking history	5 (55.6)	17 (54.8)	0.893
Body mass index	26.2 ± 5.9	26.5 ± 4.0	0.850
Duration of symptoms	8.0 ± 11.0	13.0 ± 20.4	0.492
ECOG 0	7 (77.8)	25 (80.6)	0.720
Diabetes mellitus	0 (0.0)	2 (6.5)	0.434†
Hypertension	3 (33.3)	11 (35.5)	0.850
Complete resection	6 (66.7)	22 (71.0)	0.804
***Tumor stage***			
T1	2 (22.2)	13 (41.9)	0.348
T2	7 (77.8)	14 (45.2)	
T3	0 (0.0)	2 (6.5)	
Tumor grade (high)	9 (100)	24 (77.4)	0.292
***Histological type***			
Urothelial carcinoma	7 (77.8)	28 (90.3)	0.019
Squamous cell carcinoma	2 (22.2)	0 (0.0)	
Tumor size (cm)	5.89 ± 2.60	5.46 ± 2.43	0.708
BCG therapy	0 (0.0)	4 (12.9)	0.256†
Radiotherapy	1 (11.1)	3 (9.7)	0.900†

***Footnotes:*** Univariate analyses were restricted to patients with available recurrence outcome data. Of the total cohort of 70 patients, 40 had documented recurrence status (9 with recurrence and 31 without recurrence), while 30 patients had missing data or were lost to follow-up. Values are presented as n (%) unless otherwise specified. Continuous variables are expressed as mean ± standard deviation (SD). p-values were derived using the Chi-square test or independent-samples t-test. † Fisher’s exact test was applied where expected cell counts were <5. A p-value < 0.05 was considered statistically significant.

Histological subtype showed a statistical signal in univariate analysis, with squamous cell carcinoma appearing more frequently among patients who developed recurrence compared with those who did not (p = .019). However, this finding was based on only four SCC cases and should therefore be interpreted cautiously. No significant associations were observed for age, smoking status, comorbidities, tumor stage, tumor grade, completeness of resection, or adjuvant treatments.

Because recurrence events were few (n = 9) and follow-up duration varied substantially among patients, the analysis should be interpreted as exploratory rather than confirmatory.

## DISCUSSION

This single-center cohort study explored factors associated with post-radical cystectomy recurrence in patients with bladder cancer, most of whom had muscle-invasive disease. Among patients with available follow-up data (n = 40), nine (22.5%) developed recurrence during the available follow-up period, with a mean time to recurrence of 5.0 ± 2.7 months. However, the short and heterogeneous duration of follow-up limited the ability to capture late recurrence events. In addition, the small number of recurrence events restricted the ability to identify reliable predictors, and the statistical analyses should therefore be considered exploratory.

The baseline cohort (N = 70) consisted predominantly of male patients (77.1%) in their late fifth decade of life, which is consistent with regional and international epidemiological patterns of bladder cancer. These findings are comparable to those reported by Naeem et al.^11^, who also observed a predominance of male patients and hematuria as the most common presenting symptom among bladder cancer patients in central Punjab.

A large proportion of patients in our cohort had high-grade tumors (80%) and muscle-invasive disease (T2–T4, 60%), reflecting the clinical profile of patients undergoing radical cystectomy. This may indicate relatively late presentation in our population, a pattern that has also been described in other South Asian studies. Among the variables examined, histological subtype showed a possible signal in the exploratory analysis. Squamous cell carcinoma appeared more frequently among patients who developed recurrence; however, this observation was based on only four SCC cases, two of whom experienced recurrence. Therefore, the finding should be interpreted cautiously and should not be considered evidence of a predictive relationship. Nevertheless, previous literature suggests that non-urothelial histological variants may be associated with more aggressive tumor behavior. This observation should therefore be considered hypothesis-generating and requires confirmation in larger studies.

These observations differ from those reported by Calkins et al.^12^, who found that variant histology was not independently associated with recurrence or progression in patients with high-risk non-muscle-invasive bladder cancer treated with intravesical BCG. The differences may be due to the variation in the study population and treatment approaches, as the present study population predominantly comprised patients with muscle-invasive bladder cancer undergoing radical cystectomy, whereas non-muscle-invasive bladder cancer patients undergoing bladder-sparing treatment were included in the other studies.

The previously identified prognostic factors for predicting recurrence following radical cystectomy, such as pathological stage, tumor grade, lymph node status, and tumor size, were identified in other studies. For instance, Del Giudice et al.^13^ found that pathological stage, tumor grade, and pathological upstaging are prognostic factors for cancer outcomes. However, these prognostic factors were not found to be significantly associated with cancer recurrence in the present study, likely due to the small sample size and low number of cancer recurrences.

The present study found neoadjuvant chemotherapy to be administered to 37.1% of patients, mostly gemcitabine-based regimens. Although previous studies from the United Kingdom and South India found neoadjuvant chemotherapy to improve cancer outcomes,^14^ no such significant association was found in the present study, likely due to the small sample size and lack of data regarding treatment response. Another important methodological consideration is the different lengths of follow-up for patients with and without recurrence. Patients with recurrence had a significantly shorter follow-up duration compared to those without recurrence. In addition, 42.9% of the patients had insufficient follow-up data for the assessment of recurrence. These factors are likely to have resulted in the failure to detect late recurrence cases, and hence the percentage of patients with recurrence should not be used to determine the risk of recurrence after radical cystectomy.

### Limitations:

It is a retrospective study with a small sample size and short follow-up duration. In addition, there was significant loss to follow-up and treatment variability. These factors limited the ability to perform appropriate statistical modeling and to determine independent predictors of recurrence. Despite these limitations, the study is important because it is based on real-world data from a tertiary care facility in Pakistan, and there is limited evidence on outcomes after radical cystectomy for patients from the region.

## CONCLUSION

This exploratory single-center study describes early recurrence patterns following radical cystectomy for bladder cancer. Due to the small sample size, limited and heterogeneous follow-up duration, and substantial loss to follow-up, reliable predictors of recurrence could not be established in this cohort. Therefore, the findings should be interpreted cautiously and considered hypothesis-generating rather than definitive. Future studies with larger patient populations, longer follow-up, and multicenter recruitment are needed to validate these observations and to better characterize recurrence patterns and potential risk factors following radical cystectomy.

### Recommendations:

Future research with larger patient populations, multicenter collaboration, and longer standardized follow-up is needed to better characterize recurrence patterns and potential risk factors following radical cystectomy for muscle-invasive bladder cancer.

### Author’s Contribution:

**NBN, SI, AA, AUR, SM and NZ:** Conceived, designed and did statistical analysis & editing of manuscript, is responsible for integrity of research.

**AMA and HA:** Did data collection and manuscript writing, analysis, interpretation.

**NBN, SI, AA, AUR, SM and NZ:** Did review and final approval of manuscript.
